# Reproducibility and comparative validity of a food frequency questionnaire for Australian children and adolescents

**DOI:** 10.1186/1479-5868-6-62

**Published:** 2009-09-11

**Authors:** Jane F Watson, Clare E Collins, David W Sibbritt, Michael J Dibley, Manohar L Garg

**Affiliations:** 1 School of Health Sciences, Faculty of Health, University of Newcastle, Newcastle NSW, Australia; 2 School of Medicine and Public Health, Faculty of Health, University of Newcastle, Newcastle NSW, Australia; 3 School of Public Health and The George Institute for International Health, Faculty of Medicine, University of Sydney, Sydney NSW, Australia; 4 School of Biomedical Sciences, Faculty of Health, University of Newcastle, Newcastle NSW, Australia

## Abstract

**Background:**

Dietary intake during childhood and adolescence is of increasing interest due to its influence on adult health, particularly obesity, cardiovascular disease and diabetes. There is a need to develop and validate dietary assessment methods suitable for large epidemiologic studies of children and adolescents. Limited large scale dietary studies of youth have been undertaken in Australia, due partly to the lack of a suitable dietary intake tool. A self-administered, semi-quantitative food-frequency questionnaire (FFQ), the 'Australian Child and Adolescent Eating Survey' (ACAES), was developed for youth aged 9-16 years. This study evaluated reproducibility and comparative validity of the ACAES FFQ using assisted food records (FRs) as the reference method.

**Methods:**

The ACAES FFQ was completed twice (FFQ1 and FFQ2) at an interval of 5 months, along with four one-day assisted FRs. Validity was evaluated by comparing the average of the FRs with FFQ2 (n = 113) as well as with the average of FFQ1 and FFQ2 (n = 101). Reproducibility was evaluated by comparing FFQ1 and FFQ2 (n = 101). The two methods were compared using correlations, Kappa statistics and Bland-Altman plots.

**Results:**

Correlation coefficients for comparative validity ranged from 0.03 for retinol to 0.56 for magnesium for transformed, energy-adjusted, deattenuated nutrient data, with correlation coefficients greater than 0.40 for total fat, saturated fat, monounsaturated fat, carbohydrate, sugars, riboflavin, vitamin C, folate, beta-carotene, magnesium, calcium and iron. Correlation coefficients for reproducibility ranged from 0.18 for vitamin A to 0.50 for calcium for transformed, energy-adjusted, deattenuated nutrient data. The ACAES FFQ ranked individuals reasonably accurately, with the comparative validity analysis showing that over 50% of participants were classified within one quintile for all nutrients, with only a small percentage grossly misclassified (0-7%).

**Conclusion:**

The ACAES FFQ is the first child and adolescent specific FFQ available for ranking the dietary intakes of Australian children and adolescents for a range of nutrients in epidemiologic research and public health interventions.

## Background

Developing efficient, cost-effective and valid tools for assessing the dietary intakes of children and adolescents are key research priorities [1,2]. The accurate assessment of dietary intake is critical to understanding the associations between food intake and obesity [3] as well as the influence of childhood dietary intake on chronic disease risk in adulthood [4-6]. The prevalence of obesity further necessitates the need for tools to assist in monitoring food consumption patterns. Ten per cent of the world's school-aged children are overweight or obese, with dramatically higher rates in developed regions, and prevalence continues to rise rapidly worldwide [7]. In New South Wales, Australia, the prevalence of overweight and obesity in children and adolescents doubled between 1985 and 1997 [8] with recent estimates at 25% [9].

Dietary assessment presents an ongoing challenge, particularly in large populations, with specific challenges for assessing the intakes of children and adolescents [10-12]. Although 24-hour recalls and food records (FRs) have been used successfully, the time and economic constraints of these methods make them unsuitable for most large scale studies [13]. Food frequency questionnaires (FFQs) have a lower respondent burden, are less time-consuming, less intrusive, relatively inexpensive and do not require trained interviewers, rendering them more practical for large-scale epidemiologic studies [14].

There has been limited nutrition data collected in large, representative samples of Australian children and adolescents. Only three national surveys have been conducted in the past 50 years: the 1985 National survey of schoolchildren (aged 10-15 years) [15], the 1995 National Nutrition Survey [16] which included children aged 2-18 years and the 2007 Australian National Children's Nutrition and Physical Activity Survey (ages 2-16 years) [17]. Yet the long-term nature of the development of diet-related chronic disease necessitates the collection of longitudinal dietary data. Frequency data can explain much of the variation in dietary intake and FFQs provide sufficient accuracy to relate individual diets in childhood and adolescence to subsequent health outcomes [18,19]. FFQs have been used in adults to predict associations between dietary intake and disease specific mortality and morbidity including, colon cancer, heart disease and diabetes [20]. In the US, the association between diet and health outcomes has been monitored in the Growing Up Today Study (GUTS), a longitudinal study of 16,882 9-14 year olds using the Youth-Adolescent Questionnaire (YAQ). Data collected in GUTS by the YAQ FFQ has been used to investigate the relationship between BMI and dietary intake [21], BMI and snack foods [22] and has demonstrated a relationship between BMI and intake of sugar-sweetened beverages [23] and BMI and family mealtimes [24]. There is currently no widely accepted FFQ for school-aged children in Australia. The Australian Child and Adolescent Eating Survey (ACAES) was developed in response to these gaps.

The aim of this study was to assess the reproducibility and comparative validity of a semi-quantitative, self-completed FFQ designed for school-aged children aged 9 to 16 years in the Hunter region, New South Wales, Australia.

## Methods

### Development of the ACAES FFQ

The ACAES FFQ is based on the YAQ, validated in the US by Rocket et al. [25]. Focus groups and pre-testing were undertaken with students aged 9-16 years to develop a comprehensive food list and refine the format of the FFQ. Five focus groups were held with a representative sample of 61 students: 31 primary students (9-12 years) and 30 secondary students (13-16 years), from two primary and two secondary schools. Students were asked what they usually ate for breakfast, snacks, lunch and dinner. A list was compiled and they were asked to add to this list until all the food and drinks usually consumed were included. Focus group results were used to modify the food list of the YAQ to reflect the Australian food supply, the local vernacular and the foods frequently consumed by the target population.

Once the food list was developed, a draft of the ACAES FFQ was pre-tested with a convenience sample of 41 primary and secondary students. Students were asked to complete the FFQ and circle anything that was not clear. On completion of the survey, the students discussed with a research assistant, any aspects of the FFQ they did not understand. As a result of the pre-testing, small modifications were made to the food list and ranges within response categories.

The final version of the ACAES FFQ is a 120-item FFQ with 15 supplementary questions regarding age, use of vitamin supplements, food behaviours and sedentary behaviours. The FFQ is designed as a self-administered tool, to collect information about the dietary intake of 9-16 year olds over the previous 6 months. An individual response for each food, or food type, is required, with frequency options ranging from 'Never' to '4 or more times per day', but varied depending on the food. The ACAES is semi-quantitative with a standard portion size provided for each food item and determined using 'natural' serving size (eg. slice of bread). In the absence of a natural serving size, portion sizes were derived from the 1995 National Nutrition Survey (NNS) (unpublished data purchased from the ABS). These data were for two age groups: 9-13 years and 13-16 years for boys and girls separately. Although boys had larger serve sizes than girls and the older age group had larger serve sizes than the younger, the differences were small. The average portion size for each item was calculated and used for all participants. This was the approach used by Rockett et al in the development of the Youth Adolescent Questionnaire (YAQ) [26] on which the ACAES FFQ was modelled. There were eight items without a 'natural' serving size or NNS data. For these foods, either FoodWorks 'Unspecified' serve sizes were used (5 items) or packet serve sizes (3 items). For composite items (those including more than one food) the NNS data was used and the serving size was weighted according to the NNS consumption data so that the foods consumed by the largest numbers of this age group were weighted more heavily. Due to the seasonal availability of some fruits, a separate section was included in the FFQ for seasonal fruit. The availability of seasonal fruit was determined by obtaining supermarket literature and contacting Sydney Markets, the largest fruit and vegetable market in New South Wales, which is 180 kilometres from Newcastle. Both sources indicated the number of months each year that seasonal fruit was available. The frequency categories for seasonal fruit were listed as for other food items and participants were asked to answer the question based on intake when the fruit is available. The average daily intake of seasonal fruit was calculated by adjusting for the number of months per year the fruit was available.

### Participants for the comparative validity and reproducibility study

The protocol for this study was approved by the University of Newcastle Human Research Ethics Committee (Approval No. H-498-0203) and the NSW Department of Education and Training (SERAP No 03.48). A total of 224 students in years 4, 6, 8 and 10 (aged 9-16 years) from seven schools in the Hunter region, New South Wales, Australia, were invited to participate in the study. Two rural primary schools, two urban primary schools, two rural secondary schools and one urban secondary school were included. Socio-economic status was measured using the Socio-Economic Indexes for Areas (SEIFA) allocated on the basis of school postcode and compared to the NSW average for the Index of Relative Socio-Economic Advantage/Disadvantage [27].

### Collection of dietary data

**Figure 1:**
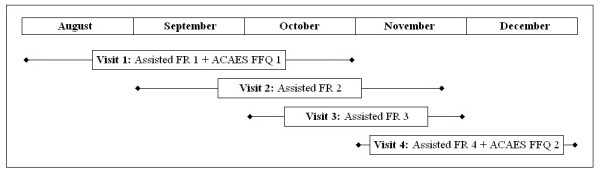
**Timeline for data collection of four assisted food records (FR) and two ACAES FFQs for the comparative validation and reproducibility of the ACAES FFQ in students aged 9-16 years in the Hunter region, 2003**.

The ACAES FFQ was administered at the beginning and end of the five month study period (Figure 1). The reference method of four assisted food records (FRs) was undertaken during the same period. The protocol was designed to capture weekly and seasonal variation.

The ACAES FFQ was completed by the children and adolescents, at school. Blank forms for the FRs were distributed to participants at school two school days prior to each data collection visit. The students were asked to complete the FR for the following day (or the day after if it was to be recorded for a Sunday). When the research team visited the school two school days later, the completed FRs were reviewed individually with the participants to clarify all food types and quantities. Any participants that had not completed a FR, instead completed a 24-hour recall (37% of records) with a research assistant following a standardised proforma. The FRs and 24-hour recalls were completed for weekdays (75% of records) and weekends (25% of records) to capture differences in eating habits. To help participants give accurate portion sizes, incremental cup and spoon measures, glasses and a ruler were used. Data from FFQ1, FFQ2 and the FRs/24-hour recalls were entered and nutrient intakes computed in FoodWorks (Version 3.02.581) using the following databases: Australian AusNut 1999 database (All Foods) Revision 14 and AusFoods (Brands) Revision 5 [28].

### Data cleaning

A total of 63 FFQs and 10 FRs were excluded from the analysis due to implausible energy intakes. The cut-offs were less than 2,090 kilojoules or greater than 20,900 kilojoules for an individual record, as used in previous studies [25].

The distribution of intakes of all nutrients, except energy, were examined for extreme values using scatterplots generated for the average nutrient values of the FRs plotted against the average of FFQ1 and FFQ2. The original FRs and/or FFQs for these participants were checked and a decision made about whether or not to exclude the record from the analysis. The two main reasons for exclusion were more than 20 main meals a week reported on the FFQ (n = 5) and food records where intakes were reported to be 'less than usual' for 3 or 4 food records (n = 2).

FFQ records were excluded if more than 10% (12 items) of the questions did not have a response (n = 36), with the number of missing items ranging from 12 - 112. Food record data was not included in the analysis if participants had completed less than three FRs. A total of 61 food records were excluded from the analysis, for 37 participants that completed less than three food records.

### Statistical Analysis

All statistical analysis was performed using STATA version 8 (2003, Stata Corporation, College Station, TX, USA) [29].

The raw data for most nutrients were not normally distributed but were positively skewed, as commonly found with dietary data [30]. Statistical and graphical assessment was used to determine the most appropriate transformation to achieve normal distribution. Logarithmic and square root transformations were attempted, with the square root transformation producing the most normalised distributions for all but a few nutrients. For simplicity, the square root transformation was used for all nutrients [30]. The nutrients were energy-adjusted using the method of Willett and Stampfer [31].

As recommended [19,32-34], comparative validity of the FFQ was evaluated by comparing the average of the FRs to FFQ2 as well as to the average of FFQ1 and FFQ2. Reproducibility was assessed by comparing the nutrient intake data from FFQ1 and FFQ2.

Means, standard deviations, medians and interquartile ranges were calculated for 22 nutrients. Spearman correlation coefficients were calculated using the unadjusted nutrient data. Pearson correlation coefficients were calculated on the data after statistical adjustment (transformation, energy-adjustment and de-attenuation). To adjust for within-person variation in the FRs, deattenuated correlation coefficients were calculated [35]. Misclassification error was assessed using the Kappa statistic [36] to compare classification of nutrient data into quintiles. In most applications, the number of categories used for calculating kappa statistics varies from two to five [37,38]. In validation studies of dietary intake, tertiles [39], quartiles [40] and quintiles [41] have all been used in the calculation of kappa statistics. The proportion grossly misclassified was also estimated.

The Bland Altman method was used to evaluate the agreement between the FFQ and FRs and between FFQ1 and FFQ2 [42]. The Bland Altman method plots the individual differences between the two measurements against the mean of the measurements. For comparative validity, the difference between the average of FFQ1 and FFQ2 and the average of the food records [FFQavg-FRavg]s was plotted on the *y-*axis and the mean of the FFQ1/FFQ2 average and the FR average [(FFQavg-FRavg)/2] was plotted on the *x-*axis. For reproducibility, the difference between FFQ1 and FFQ2 [FFQ1-FFQ2] was plotted on the *y-*axis and the average of FFQ1 and FFQ2 [(FFQ1+FFQ2)/2] was plotted on the *x-*axis. These plots indicate the direction of bias and whether it is constant across levels of intake. The 'limits of agreement' or LOA (the mean difference ± 2SDs for the difference between the two measurements) determine whether the agreement between the two methods is acceptable [43]. The differences were checked for normality as calculation of the 95% LOA assumes differences are normally distributed [44]. Where Bland Altman plots showed a tendency for the differences to increase as the magnitude of the measurement increased, the data was log-transformed and replotted. If the relationship remained, the differences were regressed onto the means. Interpretation of the Bland-Altman results was based on the categories defined by Tang [45]. **'Good agreement' **is shown when the difference between the two measurements is approximately equal to one standard deviation of the average nutrient intake from the reference method. **'Fairly good agreement' **is when the difference between the two measurements is approximately equal to two standard deviations of the average nutrient intake from the reference method and **'Bad (or poor) agreement' **occurs if the difference between the two measurements is approximately equal to three standard deviations of the average nutrient intake from the reference method.

## Results

A total of 224 students were recruited to the study. Following data cleaning, records for 113 participants remained for the comparative validity analysis (50% of those recruited) using the assisted FRs and the FFQ2 data. Complete records for 101 participants remained for the validity analysis using the average of FFQ1 and FFQ2, with the same number for the reproducibility analysis (45% of those recruited). Table 1 reports the baseline characteristics for age, height, weight, BMI and BMI z-score for the study participants. There were no significant differences between the baseline characteristics of the participants and those excluded from the analysis.

**Table 1:** Baseline characteristics of study participants (n = 113, 36% male)

	**Baseline characteristics**	
	**Mean (SD)**	**Interquartile range**

Age (years)	11.9 (1.8)	10.5 - 13.5

Height (cm)	147.2 (27.3)	142.1 - 159.0

Weight (kg)	46.3 (15.0)	36.3 - 84.7

BMI *	20.3 (4.1)	17.5 - 22.1

BMI z-score **	0.69 (1.1)	-0.09 - 1.33

*Body Mass Index = weight (kg)/height (m)^2^

**Body Mass Index z-score = the number of standard deviations above or below the reference values - computed using the LMS method [68,69]

The SEIFA codes for all schools were below the NSW average for the Index of Relative Socio-Economic Advantage/Disadvantage [27].

The median and interquartile ranges for energy and nutrients are presented in Table 2. The median values for FFQ2 and the FRs were exactly the same for monounsaturated fat, niacin equivalent, folate and iron. For all other nutrients, the values for FFQ2 were higher than those of the FRs, with the exception of polyunsaturated fat, carbohydrate, thiamin and vitamin C. The interquartile range of most nutrients tended to be wider for the FFQ data than the FRs. The dietary intakes estimated using FFQ1 were slightly higher than for FFQ2 (data not shown). This resulted in higher median values for all nutrients for the average of FFQ1 and FFQ2 when compared to the FRs.

**Table 2:** Summary statistics for the ACAES FFQ2, the average of ACAES FFQ1 and ACAES FFQ2 and the average of (3 or 4) assisted food records (FRs)

**Nutrient**	**Average of ACAES FFQ1 & ACAES FFQ2** **(n = 101)**		**ACAES FFQ2** **(n = 113)**		**Assisted FRs** **(n = 113)**	
	**Median**	**Interquartile range**	**Median**	**Interquartile range**	**Median**	**Interquartile range**

Energy (kJ)	10,483	7,500 - 12,711	10,001	6,611 - 13,056	9,272	8,031 - 10,388

Protein (g)	96	63 - 118	91	60 - 119	80	66 - 94

Total fat (g)	88	65 - 110	86	57 - 110	83	71 - 98

Saturated fat (g)	43	28 - 52	42	26 - 52	37	31 - 46

Polyunsaturated fat (g)	8	7 - 11	8	5 - 11	9	8 - 11

Monounsaturated fat (g)	30	22 - 38	29	19 - 37	29	25 - 34

Carbohydrate (g)	323	227 - 393	278	200 - 410	279	234 - 319

Sugars (g)	188	135 - 251	176	108 - 238	139	109 - 165

Fibre (g)	26	17 - 34	22	15 - 32	18	15 - 23

Thiamin (mg)	1.8	1.3 - 2.6	1.8	1.2 - 2.4	1.9	1.4 - 2.4

Riboflavin (mg)	2.7	1.9 - 3.6	2.4	1.8 - 3.3	2.2	1.5 - 3.1

Niacin (mg)	23	16 - 29	20	14 - 30	21	17 - 25

Niacin Eqv (mg)	41	28 - 52	38	26 - 55	38	30 - 43

Vitamin C (mg)	112	83 - 183	104	72 - 158	119	70 - 176

Folate (μg)	294	230 - 431	277	189 - 381	277	219 - 345

Vitamin A (μg)	1,262	913 - 1756	1,196	794 - 1838	779	576 - 1132

Retinol (μg)	616	411 - 1,127	618	364 - 1120	440	328 - 563

Betacarotene (μg)	3,044	2,087 - 4,606	2,800	1,651 - 4,770	1,679	945 - 3504

Magnesium (mg)	360	262 - 461	329	232 - 428	254	220 - 313

Calcium (mg)	1,078	779 - 1,476	1,077	721 - 1,383	809	642 - 1043

Iron (mg)	14	10 - 17	12	8 - 17	12	9 - 14

Zinc (mg)	13	9 - 16	12	8 - 16	11	9 - 12

Table 3 shows the correlation coefficients for comparative validity.

**Table 3:** Correlation coefficients for the food frequency questionnaires (FFQ1 and FFQ2) and assisted food records (FRs) for comparative validity

**Energy & Nutrients**	**Average of FFQ1 and FFQ2 vs Assisted FRs** **(n = 101)**					**FFQ2 vs Assisted FRs** **(n = 113)**				
	**Unadjusted (crude)**	**Transformed**	**De-attenuated, transformed**	**Energy-adjusted, transformed**	**De-attenuated, Energy-adjusted, transformed**	**Unadjusted (crude)**	**Transformed**	**De-attenuated, transformed**	**Energy-adjusted, transformed**	**De-attenuated, Energy-adjusted, transformed**

Energy (kJ)	0.29 **	0.29 **	0.32	-	-	0.21 *	0.20 *	0.22	-	-

Protein (g)	0.27 *	0.27 *	0.32	0.25 *	0.29	0.15	0.15	0.18	0.22 *	0.26

Fat (g)	0.34 **	0.34 **	0.39	0.30 **	0.34	0.27 **	0.27 **	0.31	0.46 **	0.53

Sat Fat (g)	0.39 **	0.39 **	0.44	0.41 **	0.46	0.33 **	0.31 **	0.35	0.46 **	0.52

Poly Fat (g)	0.20 *	0.20 *	0.25	0.03	0.04	0.14	0.16	0.20	0.17	0.21

Mono Fat (g)	0.28 **	0.31 **	0.36	0.18	0.21	0.25 *	0.26 **	0.30	0.38 **	0.44

Carbohydrate (g)	0.25 *	0.26 *	0.29	0.26 *	0.29	0.23 *	0.22 *	0.24	0.42 **	0.47

Sugars (g)	0.25 *	0.27 *	0.30	0.22 *	0.25	0.24 *	0.27 **	0.30	0.37 **	0.41

Fibre (g)	0.44 **	0.42 **	0.48	0.37 **	0.42	0.29 **	0.29 **	0.33	0.31 **	0.35

Thiamin (mg)	0.36 **	0.41	0.45	0.31 **	0.34	0.19 *	0.23 *	0.26	0.31 **	0.35

Riboflavin (mg)	0.38 **	0.45 **	0.51	0.46 **	0.52	0.25 *	0.30 **	0.34	0.47 **	0.53

Niacin (mg)	0.28 **	0.31 **	0.36	0.21 *	0.24	0.12	0.14	0.16	0.18	0.21

Niacin equiv (mg)	0.31 **	0.29 **	0.33	0.19	0.22	0.13	0.13	0.15	0.15	0.17

Vitamin C (mg)	0.25 *	0.25 *	0.28	0.36 **	0.41	0.28 **	0.29 **	0.33	0.42 **	0.48

Folate (μg)	0.46 **	0.50 **	0.55	0.48 **	0.53	0.25 *	0.31 **	0.34	0.46 **	0.51

Vitamin A (μg)	0.28 **	0.31 **	0.36	0.25 *	0.29	0.19	0.18	0.21	0.24 *	0.27

Retinol (μg)	0.17	0.22 *	0.25	0.03	0.03	0.09	0.08	0.09	0.03	0.03

Betacarotene (μg)	0.27 *	0.36 **	0.42	0.41 **	0.48	0.29 **	0.30 **	0.34	0.39 **	0.45

Magnesium (mg)	0.38 **	0.40 **	0.44	0.50 **	0.55	0.28 **	0.28 **	0.31	0.51 **	0.56

Calcium (mg)	0.43 **	0.47 **	0.52	0.51 **	0.56	0.35 **	0.37 **	0.41	0.46 **	0.51

Iron (mg)	0.44 **	0.42 **	0.46	0.39 **	0.43	0.34 **	0.33 **	0.37	0.46 **	0.51

Zinc (mg)	0.28 **	0.26 *	0.31	0.25 *	0.30	0.18	0.15	0.18	0.27 **	0.32

**Measure of central tendency**	**0.285 §**	**0.34 §§**	**0.38 §§**	**0.30 §§**	**0.34 §§**	**0.245 §**	**0.24 §§**	**0.27 §§**	**0.34 §§**	**0.39 §§**

Spearmans correlations undertaken in unadjusted (crude) data. Pearsons correlations undertaken on all other data (square-root transformed).

* denotes p-value < 0.05; ** denotes p-value < 0.01; **§ **denotes median; **§§ **denotes mean.

When the unadjusted data for FFQ2 and the average of the FRs was compared, Spearman's correlation coefficients ranged from 0.09 (retinol) to 0.35 (calcium). The median correlation for the unadjusted nutrient intakes of the two methods was 0.25. After transformation, the mean Pearson correlation was 0.24, ranging from 0.13 (niacin equivalent) to 0.37 (calcium). Deattenuation of the correlations for the transformed data increased the mean correlation to 0.27, ranging from 0.03 (retinol) to 0.41 (calcium). When the transformed data was energy-adjusted, the mean correlation coefficient increased to 0.34, with substantially larger coefficients for most nutrients except retinol, niacin equivalent, fibre and polyunsaturated fat. Deattenuation of the correlations for the transformed, energy-adjusted data increased the mean correlation to 0.39, ranging from 0.17 (niacin equivalent) to 0.56 (magnesium). The mean correlation for FFQ1 and the average of the FRs was higher than for FFQ2 for the crude data, the transformed data and the de-attenuated, transformed data (0.32, 0.34 and 0.38, respectively). However, the correlations for FFQ1 and the average of the FRs dropped following energy adjustment, resulting in lower correlations (data not shown) than for the energy-adjusted data for FFQ2.

Reproducibility was evaluated by calculating correlation coefficients for FFQ1 and FFQ2, as shown in Table 4. Spearman correlations for the unadjusted data ranged from 0.34 (sugars) to 0.53 (niacin equivalent). The median correlation for the unadjusted nutrient intakes of the two FFQs was 0.46. After transformation, the mean correlation using Pearson correlation coefficients was lower (0.44), ranging from 0.34 (sugars) to 0.51 (niacin equivalent). When the transformed data was energy-adjusted, the mean correlation coefficient decreased from 0.44 to 0.32, with substantially smaller coefficients for all nutrients, except magnesium and calcium which increased.

**Table 4:** Correlation coefficients for the ACAES FFQ1 and ACAES FFQ2 for reproducibility

**Energy & Nutrients**	**FFQ1 vs FFQ2 (n = 101)**		
	**Unadjusted (crude)**	**Transformed**	**Energy-adjusted, transformed**

Energy (kJ)	0.45 **	0.44 **	-

Protein (g)	0.50 **	0.48 **	0.36 **

Fat (g)	0.49 **	0.50 **	0.31 **

Sat Fat (g)	0.48 **	0.49 **	0.35 **

Poly Fat (g)	0.44 **	0.44 **	0.33 **

Mono Fat (g)	0.50 **	0.51 **	0.33 **

Carbohydrate (g)	0.37 **	0.35 **	0.20*

Sugars (g)	0.34 **	0.34 **	0.31 **

Fibre (g)	0.44 **	0.40 **	0.31 **

Thiamin (mg)	0.40 **	0.37 **	0.21*

Riboflavin (mg)	0.46 **	0.43 **	0.21*

Niacin (mg)	0.52 **	0.50 **	0.36 **

Niacin equiv (mg)	0.53 **	0.51 **	0.41 **

Vitamin C (mg)	0.45 **	0.38 **	0.34 **

Folate (μg)	0.45 **	0.41 **	0.28 **

Vitamin A (μg)	0.42 **	0.37 **	0.18

Retinol (μg)	0.46 **	0.36 **	0.20

Betacarotene (μg)	0.50 **	0.48 **	0.45 **

Magnesium (mg)	0.46 **	0.44 **	0.49 **

Calcium (mg)	0.46 **	0.48 **	0.50 **

Iron (mg)	0.45 **	0.41 **	0.29 **

Zinc (mg)	0.49 **	0.49 **	0.40 **

**Measure of central tendency**	**0.46 §**	**0.44 §§**	**0.32 §§**

Spearmans correlations undertaken in unadjusted (crude) data.

Pearsons correlations undertaken on all other data (square-root transformed).

* denotes p-value < 0.05; ** denotes p-value < 0.01; **§ **denotes median; **§§ **denotes mean.

Table 5 shows the results of the kappa analysis undertaken as part of the comparative validity.

**Table 5:** Kappa statistics for food-frequency questionnaires (FFQ1 and FFQ2) and assisted food records (FRs) for comparative validity

**Energy & Nutrients**	**Average FFQ1 and FFQ2 vs FRs** **(n = 101)**					**FFQ2 vs FRs** **(n = 113)**				
	**Weighted kappa**	**% correctly classified into same quintile**	**% classified within one quintile**	**% grossly misclassified**	**Strength of agreement^a^**	**Weighted kappa**	**% correctly classified into same quintile**	**% classified within one quintile**	**% grossly misclassified**	**Strength of agreement^a^**

Energy (kJ)	0.27 **	30	58	3	Fair	0.14	22	56	4	Slight

Protein (g)	0.22 *	27	57	5	Fair	0.13	20	56	6	Slight

Fat (g)	0.25 *	26	59	3	Fair	0.21 *	21	58	3	Fair

Sat Fat (g)	0.36 **	33	64	3	Fair	0.31 **	27	62	3	Fair

Poly Fat (g)	0.20 *	32	61	5	Slight	0.11	28	58	6	Slight

Mono Fat (g)	0.21 *	27	60	5	Fair	0.20 *	22	58	4	Slight

Carbohydrate (g)	0.23 *	23	56	2	Fair	0.21 *	27	56	4	Fair

Sugars (g)	0.30 **	30	61	4	Fair	0.24 *	31	58	4	Fair

Fibre (g)	0.40 **	37	68	1	Fair	0.25 **	21	60	2	Fair

Thiamin (mg)	0.29 **	28	55	1	Fair	0.21 *	27	59	4	Fair

Riboflavin (mg)	0.37 **	27	66	1	Fair	0.22 *	21	58	1	Fair

Niacin (mg)	0.27 **	29	63	4	Fair	0.13	26	55	5	Slight

Niacin equiv (mg)	0.33 **	23	63	4	Fair	0.16	24	57	6	Slight

Vitamin C (mg)	0.23 *	25	64	5	Fair	0.27 **	30	61	3	Fair

Folate (μg)	0.41 **	29	60	0	Moderate	0.22 *	19	58	2	Fair

Vitamin A (μg)	0.24 *	34	56	3	Fair	0.14	21	56	7	Slight

Retinol (μg)	0.12	18	52	12	Slight	0.09	20	52	4	Slight

Betacarotene (μg)	0.24 *	27	64	4	Fair	0.27 **	28	65	6	Fair

Magnesium (mg)	0.35 **	29	64	2	Fair	0.26 **	27	61	5	Fair

Calcium (mg)	0.35 **	32	71	5	Fair	0.31 **	29	65	5	Fair

Iron (mg)	0.45 **	27	70	1	Moderate	0.36 **	27	64	2	Fair

Zinc (mg)	0.24 *	23	56	1	Fair	0.17 *	19	51	4	Slight

Kappa statistics undertaken on unadjusted (crude) data.

* denotes p-value < 0.05; ** denotes p-value < 0.01

^a ^Landis and Koch classification (Landis and Koch 1977)

For the comparative validity analysis, the weighted kappa values for the nutrient intake data from FFQ2 and the average of the FRs ranged from 0.09 (retinol) showing 'slight' agreement, to 0.36 (iron), showing 'fair' agreement. All other nutrients showed 'slight' to 'fair' agreement. The proportion of individuals correctly classified into the same quintile was highest for sugars (31%) and lowest for zinc and folate (19%). The percentage classified within one quintile was highest for beta-carotene and calcium (65%) and lowest for zinc (51%). The percentage grossly misclassified (those ranked in the lowest quintile for the FFQ data but the highest quintile for assisted FRs) was small (1-7%).

For the reproducibility analysis of the nutrient intake data from FFQ1 and FFQ2, niacin (0.54) showed the strongest weighted kappa value and was classified as having 'moderate' agreement, while sugars had the lowest weighted kappa value (0.36) indicating 'fair' agreement. As shown in Table 6, with the exception of carbohydrate, sugars and vitamin A, all nutrients showed 'moderate' agreement with values between 0.41 and 0.60. The proportion of individuals correctly classified into the same quintile was highest for niacin equivalent (39%) and lowest for sugars (23%). The percentage classified within one quintile was highest for protein (79%) and lowest for vitamin A (63%). A very small percentage of individuals were grossly misclassified, ranging from none for Vitamin C and 5% for fibre.

**Table 6:** Kappa statistics for food-frequency questionnaires (FFQ1 and FFQ2) for reproducibility

**Energy & Nutrients**	**FFQ1 vs FFQ2** **(n = 101)**				
	**Weighted kappa**	**% correctly classified into same quintile**	**% classified within one quintile**	**% grossly misclassified**	**Strength of agreement^a^**

Energy (kJ)	0.42 **	30	69	1	Moderate

Protein (g)	0.48 **	30	79	4	Moderate

Fat (g)	0.47 **	34	71	1	Moderate

Sat Fat (g)	0.43 **	32	72	3	Moderate

Poly Fat (g)	0.44 **	37	69	4	Moderate

Mono Fat (g)	0.45 **	29	74	2	Moderate

Carbohydrate (g)	0.37 **	30	67	3	Fair

Sugars (g)	0.36 **	23	68	4	Fair

Fibre (g)	0.44 **	34	73	5	Moderate

Thiamin (mg)	0.41 **	34	66	4	Moderate

Riboflavin (mg)	0.41 **	30	68	4	Moderate

Niacin (mg)	0.54 **	36	77	2	Moderate

Niacin equiv (mg)	0.53 **	39	77	3	Moderate

Vitamin C (mg)	0.42 **	32	67	0	Moderate

Folate (μg)	0.41 **	36	72	4	Moderate

Vitamin A (μg)	0.38 **	36	63	3	Fair

Retinol (μg)	0.47 **	36	72	2	Moderate

Betacarotene (μg)	0.49 **	33	70	1	Moderate

Magnesium (mg)	0.41 **	28	70	4	Moderate

Calcium (mg)	0.45 **	27	68	3	Moderate

Iron (mg)	0.41 **	28	66	3	Moderate

Zinc (mg)	0.49 **	32	75	3	Moderate

Kappa statistics undertaken on unadjusted (crude) data.

* denotes p-value < 0.05; ** denotes p-value < 0.01

^a ^Landis and Koch classification (Landis and Koch 1977)

When applying the Bland Altman method to the comparative validity data, the mean difference between the methods using the raw nutrient intake data was positive for most nutrients, indicating that when compared to FRs, the ACAES FFQ provides higher estimates for the intake of all nutrients, except polyunsaturated fat and thiamin.

**Figure 2:**
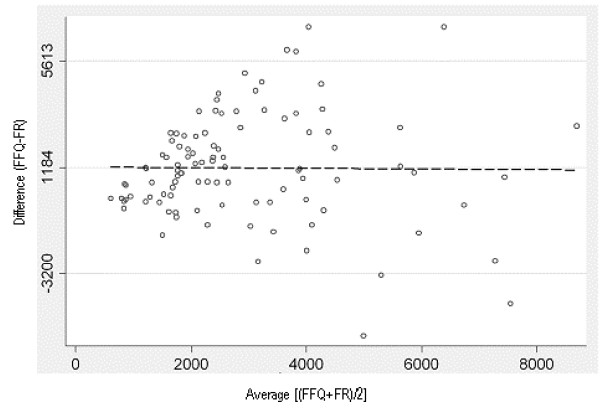
**Bland Altman plot of the difference between beta-carotene intake measured by FFQ1 and FFQ2 and the average of the assisted 24-hr recalls, plotted against the mean beta-carotene intake of the two methods, for the comparative validity analysis**. The solid horizontal line in the centre indicates the mean difference between the two methods (1184 μg) and the solid lines above and below this indicate ± 2SDs (-3200 μg to 5613 μg)

Figure 2 shows the Bland-Altman plot for comparative validity analysis using the raw data for beta-carotene. The plot is scattered, indicating the bias is constant across levels of intake. The limits of agreement are approximately equal to two standard deviations of the FR data, showing fairly good agreement [45] between the FRs and the average of the FFQs.

**Figure 3:**
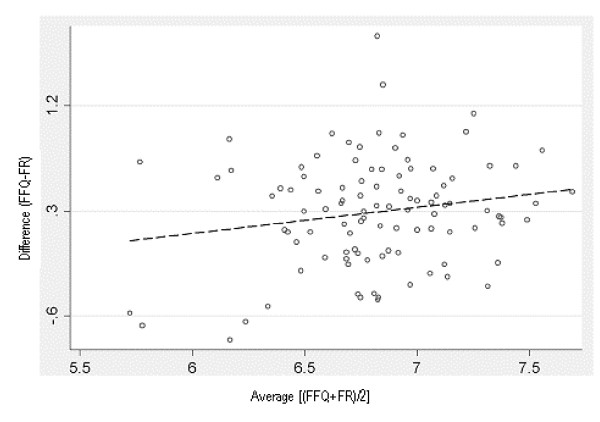
**Bland Altman plot of the difference between log-transformed data of calcium intake measured by FFQ1 and FFQ2 and the average of the assisted 24-hr recalls, plotted against the mean calcium intake of the two methods, for the comparative validity analysis**. The solid horizontal line in the centre indicates the mean difference between the two methods (34% greater for the FFQ - antilog) and the solid lines above and below this indicate ± 2SDs (-53% to 336% - antilog).

The Bland Altman plots showed a tendency for the differences to increase as the magnitude of measurement increased for all nutrients, except beta-carotene, polyunsaturated fat, thiamin and vitamin C. The data for nutrients showing this trend were log transformed in an attempt to remove this relationship [42,44]. For calcium, folate, sugars, riboflavin, thiamin and vitamin A this created a plot with a more consistent bias across levels of intake as shown in Figure 3 for calcium. However, the wide 95% LOA (antilog values of -53% to 336%) indicated discrepancies between the two methods for some individuals.

Log-transformation did not remove this relationship for energy, protein, total fat, saturated fat, monounsaturated fat, carbohydrate, fibre, niacin, retinol, iron and zinc. For these nutrients, the absolute residuals from a fitted regression line method was used [44] and this regression analysis showed significant relationships existed for these nutrients.

Bland Altman analysis of the reproducibility data showed a small, positive mean difference for all nutrients, indicating that FFQ1 tended to give slightly higher estimates of nutrient intake than FFQ2. The plots for all nutrients were scattered, showing consistent bias across all levels of intake. The LOA were wide (greater than ± 2SDs of the assisted FRs) indicating poor agreement between the two FFQs across the range of intakes.

## Discussion

Comprehensive FFQs have been developed to assess the total diets of children and adolescents in the United States [26], Norway [46], the United Kingdom [47] and Italy [48] with a limited number of validation studies undertaken. These studies demonstrated that for these populations, dietary intakes could be measured reasonably well using an FFQ. Differences in study methods, populations and between-person variation make it difficult to compare validity across studies internationally. However, it is worth noting that the results from the present study are similar to those of previous FFQ validation analyses for children and adolescents [25,47,49-53].

The validity of the ACAES FFQ was assessed by comparing the nutrient estimates from the average of the FRs with ACAES FFQ2 as well as comparing the average of the FRs with the average of ACAES FFQ1 and ACAES FFQ2. Although ACAES FFQ2 represents the conceptually appropriate time sequence, the process of keeping FRs might alter awareness of food intake and artificially improve accuracy in completing it [19]. However, comparing ACAES FFQ1 with the FRs, would tend to underestimate validity because ACAES FFQ1 asked about diet prior to the study period. Therefore, the use of the average of ACAES FFQ1 and ACAES FFQ2 provides a combination of minimal and maximal estimates and is likely to be a more accurate indication of true validity [19].

Food frequency questionnaires tend to estimate higher nutrient intakes when compared to 24-hour recalls and FRs [54]. This was shown in the present study, with the ACAES FFQ providing higher estimates of intakes for all nutrients, except polyunsaturated fat, carbohydrate, thiamin, niacin and vitamin C, when compared to assisted FRs.

The correlation results from this study are comparable to those for the YAQ [25]. This was anticipated given that ACAES was modified from the YAQ and both studies had a similar design. When the correlation results for relative validity of the ACAES FFQ are compared to the YAQ [25], the correlation for carbohydrate is equivalent (r = 0.47), while the correlations for fat, saturated fat, folate, iron and riboflavin (Vitamin B2) are marginally lower. The correlations for beta-carotene, magnesium and zinc were higher for the ACAES FFQ compared to the YAQ. Correlations for all other nutrients were either lower (energy, protein, monounsaturated fat, fibre, thiamin, vitamin C, vitamin A, calcium), not significant (polyunsaturated fat, niacin and retinol) or not common to both studies (sugars, niacin equivalent).

The mean correlation coefficients for the common nutrients between both studies (without vitamin supplementation) were calculated, excluding those nutrients in our study with correlation coefficients that were not significant (>0.05) (polyunsaturated fat, niacin and retinol). Rockett observed a mean correlation of 0.42 between the 2nd YAQ and the mean of three 24-hour recalls [25], compared to 0.39 for this study, indicating similar correlations between the two methods in both studies.

When compared to the correlations for other comparative validity studies, the ACAES FFQ showed similar results. The ACAES FFQ had higher correlations than the Block Kids Questionnaire (BKQ) for fibre and calcium, but not for energy [49]. Our study had almost identical correlations for calcium as two FFQs designed specifically to assess calcium intake in children and adolescents in the US and Italy [50,48,51]. As found in the present study, others have also shown poor agreement for vitamin A [32], polyunsaturated fat [52,55] and protein [47,53]. This is probably due to the large day-to-day variation in the intakes of these nutrients by children and adolescents, particularly girls. To reflect the *usual *intakes of vitamin A, polyunsaturated fat and protein, a greater number of recording days is necessary, with as many as 46 days recording required to estimate the usual vitamin A intake for females aged 5-17 years [56].

In order to detect associations between diet and disease, it is suggested that correlations need to be at least 0.3 or 0.4 [32]. The present study had significant correlations greater than 0.3 for the deattenuated data of all nutrients except protein and vitamin A.

The reproducibility results of the YAQ [26] were similar to the present study. The mean correlation for the YAQ was 0.41 for the log-transformed, energy adjusted data for the 7 nutrients presented. For the 21 nutrients examined in the ACAES FFQ reproducibility analysis, the mean correlations were 0.46 for raw data, 0.44 for transformed, 0.32 for transformed, energy-adjusted data. In the present study, the correlations for many nutrients dropped following transformation and again following energy adjustment. The square-root transformation was applied to all nutrients due to its appropriateness for the majority of nutrients for the ACAES FFQ1 and FFQ2 data. However, it was not the most statistically appropriate transformation for some nutrients in ACAES FFQ2 and is likely to be the reason the correlation coefficients for these nutrients dropped following transformation. The reduction in the reproducibility correlations following energy adjustment has occurred in other studies of reproducibility [57-60] and is likely to be due to systematic errors of over- and under-estimation between ACAES FFQ1 and FFQ2. Although we have no way to assess this directly, adjustment for energy increases correlation coefficients when the variability in nutrient intake is related to energy intake, but results in lower correlations when the nutrient variability depends on systematic errors of overestimation and underestimation [35]. The correlation coefficient for reproducibility for total fat intake in our study was higher for crude data (0.49) when compared to the reproducibility correlation (0.41) for an FFQ for children aged 2-5 years developed specifically to assess fat intake [61] demonstrating good validity for total fat intake.

Bland Altman analyses have not often been reported in studies comparing FFQs with diet records in children and adolescents [53]. In the only other study reporting Bland Altman results for children (11-13 years) [53], the findings were similar. The mean differences were positive and of very similar magnitude to the current study, with the limits of agreement wide for all nutrients presented (energy, fat, sugar, calcium and protein). Similarly, the Bland Altman plots from the raw data showed strong trends of increasing difference with increasing intakes. The Bland Altman results for the present study show that the ACAES FFQ is not suitable for estimating *absolute *intakes for children and adolescents, but is appropriate for *ranking *intakes. The similarity of results between the present study and that of Lietz et al [53], suggests that large variation in the agreement between methods may be characteristic of child/adolescent populations and that these results may be due to the usual variability of dietary intake of children and adolescents. Agreement between FFQs and 24-hour recalls or food records may be lower in children and adolescents than in adults due to the greater day to day variation in their dietary intake [56]. Adolescents have highly variable food patterns, with possibly half of the foods they eat varying greatly [62].

The reference method of choice for FFQ validation studies is weighed food records or diet records [34]. Although 24-hour recalls have less respondent burden, their sources of error tend to be more correlated with the error in an FFQ due to reliance upon memory, conceptualisation of portion sizes and distortion of reported diet [34]. There was a high proportion of children aged 9-12 years (71.7%) in the sample and it was expected that some of these participants would be less likely to complete a food record. The 24-hour recall method was undertaken with participants that had not completed their food records due to it's suitability to participants with limited cooperation or literacy [34]. Interestingly, approximately the same proportion of younger children (9-12 years) and older children (13-16 years) completed 24-hour recalls (36.8% and 38.1% respectively) because they had not completed a food record.

Standard portion sizes were applied to the ACAES FFQ because frequency has been found to be more discriminatory than portion size [63,64]. Adding open-ended questions regarding portion size can actually reduce validity of an FFQ due to sources of error in conceptualising serve sizes and large, within-person variations in serving sizes when the same food is consumed on different occasions [20].

A general limitation of validation studies is that the results are not necessarily transferable to another population [65]. A sample size of at least 50 is desirable for each demographic group [33], and ideally between 100 and 200 participants [19]. The sample size in the present study was inadequate to compare the validity and reproducibility of subsets for age, gender or BMI category. Performance of the ACAES FFQ would need to be tested in populations of different SES and ethnicity.

A total of 43 participants were excluded due to implausible energy intakes reported on the ACAES FFQ, representing 19% of the number recruited. A total of 36 of these were for high intakes, while seven were for implausibly low intakes. This is a high proportion and we feel that the main reason was the completion of the ACAES FFQ in a class environment. It seemed that by completing the FFQ as a group, there may have been more distractions compared to completing it on their own or with a parent present. Some of the excluded students marked the highest intake category for almost all food items. In a subsequent study by the same research team using the ACAES FFQ with primary school children, only 2 out of 60 participants (3.3% of the sample) were excluded due to implausible energy intakes (data not shown). These children completed the FFQ independently at the school, rather than in a class environment. In future studies, it is recommended that the ACAES FFQ is completed by the child independently, rather than in a class environment.

Despite these limitations, there are many aspects of the study design and analysis that are likely to contribute to an underestimation of the true validity and reproducibility of the ACAES FFQ. Food records were used predominantly (63% of records) and combined with 24-hour recalls (37% of records). Due to differences in the biases of food records and FFQs, the predominant use of food records is likely to underestimate validation coefficients when compared to 24-hour recalls as the reference method [19]. The day of the week is also likely to influence the results of validation studies. In the present study, the proportion of records collected on weekdays (75%) and weekends (25%) is close to the actual proportion of weekdays and weekend days (71% and 29%, respectively). When comparing 14 days versus 2 days of food records in Australian children, Jenner et al found that correlation coefficients were slightly higher when both days of the two day records were taken on weekdays [66]. It is possible that this is due to a perceived 'usual' intake during the week and greater variation on weekends. Participants reported a 'usual' intake for 64% of the records collected. Of the remaining 36% of records, half were reported as 'less than usual' with the other half reported as 'more than usual'. This large proportion of 'more' or 'less' than usual intakes may be typical of the intakes of this age group, but is likely to contribute to reduced agreement with the FFQ, where they record their perceived 'usual' intake. The conservative approach to excluding records from the data analysis also contributes to underestimating the validity of the FFQ. For example, respondents with food records that were inconsistent with their FFQs were not excluded if their responses were plausible. The most extreme examples of these were intakes of liver in the FFQ, but none reported on the FR days (resulting in particularly poor agreement for vitamin A and retinol) and reporting items in their food records that were not on the FFQ (eg. oysters, popcorn, slurpee, added sugar, chocolate topping, sherbet).

Although 24-hour recalls capture rich information on food consumption, they measure episodically consumed foods poorly. Recent statistical modelling has suggested that combining FFQ data with a limited number of 24-hour recalls may provide the most accurate method of estimating usual dietary intake at the individual level, supporting the use of both methods in national surveillance [67].

## Conclusion

This study evaluates the strengths and limitations of a self-administered FFQ developed for Australian children and adolescents aged 9 to 16 years. When compared to assisted FRs, the ACAES FFQ overestimates nutrient intakes and is therefore not suitable for estimating absolute intakes for individuals in this age group. However, with the exception of polyunsaturated fat and retinol, it demonstrates an acceptable ability to correctly classify participants into quintiles of intake.

This FFQ has comparable results across a wide range of nutrients to other FFQs designed for children and adolescents evaluated for validity and reliability. It provides an important contribution to the tools available for assessing usual intakes in children and is a useful tool for estimating the dietary intakes of total fat, saturated fat, carbohydrate, sugars, fibre, vitamin C, folate, beta-carotene, calcium and iron in clinical practice, epidemiologic research and public health interventions among Australian youth and adolescents.

## Competing interests

A scannable version of the ACAES FFQ is currently under development and will be available for research application.

## Authors' contributions

JW collected the data, performed the statistical analysis and drafted the manuscript. JW, CC, DS, MD and MG developed the study, and participated in its design and coordination. CC and DS provided assistance for the statistical analysis and helped to draft the manuscript. All authors read and approved the final manuscript.
